# Chromatin Accessibility Predetermines Odontoblast Terminal Differentiation

**DOI:** 10.3389/fcell.2021.769193

**Published:** 2021-11-25

**Authors:** Qian Zhang, Zhen Huang, Huanyan Zuo, Yuxiu Lin, Yao Xiao, Yanan Yan, Yu Cui, Chujiao Lin, Fei Pei, Zhi Chen, Huan Liu

**Affiliations:** ^1^ The State Key Laboratory Breeding Base of Basic Science of Stomatology and Key Laboratory for Oral Biomedicine of Ministry of Education, School and Hospital of Stomatology, Wuhan University, Wuhan, China; ^2^ Fujian Key Laboratory of Developmental and Neuro Biology, College of Life Science, Fujian Normal University, Fuzhou, China; ^3^ Division of Rheumatology, Department of Medicine, University of Massachusetts Medical School, Worcester, MA, United States; ^4^ Department of Periodontology, School of Stomatology, Wuhan University, Wuhan, China

**Keywords:** tooth development, odontogenesis, dental mesenchymal stem cells, transcription factors, epigenetics, H3K27ac, chromatin immunoprecipitation sequencing, RNA-seq

## Abstract

Embryonic development and stem cell differentiation are orchestrated by changes in sequential binding of regulatory transcriptional factors to their motifs. These processes are invariably accompanied by the alternations in chromatin accessibility, conformation, and histone modification. Odontoblast lineage originates from cranial neural crest cells and is crucial in dentinogenesis. Our previous work revealed several transcription factors (TFs) that promote odontoblast differentiation. However, it remains elusive as to whether chromatin accessibility affects odontoblast terminal differentiation. Herein, integration of single-cell RNA-seq and bulk RNA-seq revealed that *in vitro* odontoblast differentiation using dental papilla cells at E18.5 was comparable to the crown odontoblast differentiation trajectory of OC (osteocalcin)-positive odontogenic lineage. Before *in vitro* odontoblast differentiation, ATAC-seq and H3K27Ac CUT and Tag experiments demonstrated high accessibility of chromatin regions adjacent to genes associated with odontogenic potential. However, following odontoblastic induction, regions near mineralization-related genes became accessible. Integration of RNA-seq and ATAC-seq results further revealed that the expression levels of these genes were correlated with the accessibility of nearby chromatin. Time-course ATAC-seq experiments further demonstrated that odontoblast terminal differentiation was correlated with the occupation of the basic region/leucine zipper motif (bZIP) TF family, whereby we validated the positive role of ATF5 *in vitro*. Collectively, this study reports a global mapping of open chromatin regulatory elements during dentinogenesis and illustrates how these regions are regulated via dynamic binding of different TF families, resulting in odontoblast terminal differentiation. The findings also shed light on understanding the genetic regulation of dentin regeneration using dental mesenchymal stem cells.

## Introduction

Cell fate specification is achieved through spatiotemporal gene expression during embryonic development, tissue regeneration, or cell reprogramming (Spitz and Furlong, 2012). A set of tissue-specific transcription factors (TFs) regulate these genes at the transcriptional level. They potentially recognize and interact with their specific DNA-binding motifs in the genome to drive lineage-specific gene expression at different developmental stages (Lee and Young, 2013). Moreover, TFs are master regulators in gene regulatory networks (GRNs) to establish competency for different cell fates (Davidson, 2010). However, a majority of potential DNA-binding sites are inaccessible because the genomic DNA in eukaryotic cells is occluded by higher-order chromatin structures (Luger et al., 1997). Within this context, gene regulation occurs at gene regulatory regions in the opened chromatin, which allows for the binding of TFs and functioning of RNA polymerase (Calo and Wysocka, 2013). These types of opened chromatin regions, such as active promoters or enhancers, are characterized by histone modifications that flank nucleosome-free regions, including H3K4 methylation and H3K27 acetylation (Creyghton et al., 2010). The mechanism by which TFs recognize their binding motifs or influence the chromatin accessibility to initiate different biological processes remains largely unknown.

Dental papilla cells are cranial neural crest-derived mesenchymal populations, which form odontoblasts and dentin. Numerous TFs play crucial roles in odontoblastic differentiation via the regulation of gene expression programs, such as RUNX2, DLX3, SOX9, SOX2, and KLF6 (Li et al., 2011; Wang et al., 2014; Yang G. et al., 2017; Yang Y. et al., 2017; Chen et al., 2021). In our recent studies, three zinc-finger TFs, KLF4, SALL1, and ZEB1, have been revealed to regulate odontoblastic differentiation via different mechanisms. KLF4 regulates *Dmp1* and *Sp7* transcription through modulation of histone acetylation. Interaction of SALL1 with RUNX2 directly activates TGF-β2 to regulate the commitment of odontoblast lineages. Besides, ZEB1 alters the chromatin accessibility of *cis*-elements adjacent to genes including *Runx2* in the early stage and directly promotes *Dspp* transcription in the late stage (Tao et al., 2019; Xiao et al., 2021b; Lin et al., 2021). The previous findings affirm that chromatin accessibility is associated with odontoblast terminal differentiation. However, a global view of the interaction of lineage-determining TFs with dynamic changes in chromatin accessibility during odontoblast cell fate specification is elusive.

To fill this knowledge gap, we validated that postnatal day 0 (PN0) OC (osteocalcin)-positive odontogenic lineage in the first lower molar tooth germ mainly contributes to the crown odontoblast layer, which served as a potential *in vivo* odontoblast differentiation model. We employed this *in vivo* model and the frequently adopted *in vitro* odontoblast differentiation model to comprehensively analyze the mechanism of chromatin accessibility in odontoblast terminal differentiation.

## Materials and methods

### Animal maintenance

All C57BL/6 mouse experiments used for mDPC culture and subsequent ATAC-seq and RNA-seq were performed under the guideline and approval of the Institutional Animal Care and Use Committees at the School and Hospital of Stomatology attached to Wuhan University (protocol no.S07920070I). The OC-Cre (Zhang et al., 2013) and Rosa26-mTmG (Muzumdar et al., 2007) alleles used in this study were described. All experiments involving these two lines were performed with the approval of the Institutional Animal Care and Use Committees at the School of Life Science in Fujian Normal University (protocol no. 20210007). All animal experiments were performed in accordance to the ARRIVE guidelines 2.0.

### Cell culture

We isolated primary dental papilla mesenchymal cells (mDPCs) from embryonic day 18.5 (E18.5) first molar tooth germ. A dissection needle was employed to remove the dental epithelium after digestion using 0.75 mg/ml of dispase (Becton, Dickinson and Co., Franklin Lakes, NJ, USA). The tooth germ was isolated from the mandible using forceps, dispersed, and digested with 0.25% trypsin-EDTA (Life Technologies, Carlsbad, CA, USA) at 37°C for 15 min. Cells were cultured in Dulbecco’s modified Eagle medium (DMEM; Hyclone, Pittsburgh, PA, USA). To induce mineralization, mDPCs between passage 2 and 4 were cultured in an induction medium supplemented with 50 μg/ml of ascorbic acid (Sigma, St. Louis, MO, USA), 10 mmol/L sodium-glycerophosphate, and 10 nmol/L dexamethasone (Sigma). All the cells used in this study were maintained in 5% CO_2_ at 37°C, and the medium was replenished every 2 days.

### Plasmid construction dual-luciferase assay, and transfection

For enhancer activity assay, DNA fragments synthesized by Sangon Biotech (Shanghai, China) were inserted into the pGL3-promoter vector. Phusion^®^ polymerase (NEB, USA) was used to clone the full-length of open-reading frame of ATF5, and subcloned into pcDNA3.1 (+). For dual-luciferase assay, we cotransfected mDPCs with pGL3 vector along with the pRL-TK plasmid and ATF5-OE plasmid or pcDNA3.1 (+) using Lipofectamine 2000 (Life Technologies). Four days posttransfection, the dual-luciferase assay was performed using the luciferase Assay System (Promega) following the protocol of the manufacturer. Triplicate wells were analyzed. Firefly luciferase activity from the whole-cell lysates was normalized using Renila activity internal control.

Lentivirus-expressing short-hairpin RNA (shRNA) against Atf5 (shAtf5) (top strand: GAT​CCG​CGG​GAG​ATC​CAG​TAC​GTG​AAT​TCA​AGA​GAT​TCA​CGT​ACT​GGA​TCT​CCC​GCT​TTT​TTG; bottom strand: AAT​TCA​AAA​AAG​CGG​GAG​ATC​CAG​TAC​GTG​AAT​CTC​TTG​AAT​TCA​CGT​ACT​GGA​TCT​CCC​GCG) and empty control (control) were generated from HanBio (HanBio, China). mDPC at passage was infected with lentivirus, 4 days after which knockdown efficiency was validated using qRT-PCR.

### Single-cell RNA-Seq and downstream analysis

Single-cell RNA-seq was performed on the 10x Chromium platform using Chromium Single Cell 3′ Library and Gel Bead Kit v3 (10x Genomics; Annoroad Genomics, China). Following the quality check, the DNA library was sequenced on the Illumina Novaseq 6000 (Illumina, Annoroad Genomics, China). Filtered reads were mapped to mm10 transcriptome using Cell Ranger v3.0 (10x Genomics) (Zheng et al., 2017). The Seurat package (v3.0) (Stuart et al., 2019) was employed in R for downstream analysis. Briefly, raw count matrices were filtered to remove barcodes with less than 500 genes expressed; more than 8,000 genes expressed, and a there was a high percentage of UMIs from the mitochondria (>10%), leaving 6,720 cells for the first round of clustering. Counts were normalized and scaled using the SCTransform function (Hafemeister and Satija, 2019). The first round of dimension reduction and clustering was performed using “dim = 1:30.” Dmp1+/GFP+ (GFP >1) served as a terminal odontoblast cluster for the OC-positive population. Considering the distance between other clusters and this cluster, we subset all clusters with “nearby” as odontogenic lineage for further analysis. Finally, 975 cells were picked as OC-positive odontogenic lineage. Dimension reduction and clustering were performed for the second round, and clusters were visualized using tSNE plots. We reconstructed cell differentiation trajectories using Monocle (Trapnell et al., 2014) (v3.0) from the Seurat object above. All cluster information was inherited from Seurat. Based on counts of genes in each cell, cell trajectories were imputed using the “order_cells” function. Distribution of regulons was generated in SCENIC packages (v 1.1.2) (Aibar et al., 2017) in R.

### Transposase-accessible chromatin assay with high-throughput sequencing

We cultured mDPCs in a mineralization medium and normal culture medium for 0, 3, 5, 7, and 9 days. OC-positive and OC-negative tooth germ cells were harvested as follows: The mandible of PN0 OC-Cre and Rosa26-mTmG was treated with dispase to allow for the removal of dental epithelium using dissection needles. Fine forceps were then applied to isolate the tooth germ. To obtain a single-cell suspension, tooth germ was disassociated with trypsin and subjected to FACS sorting to acquire 10^5^ OC-positive and 10^5^ OC-negative cells. ATAC-seq libraries were prepared as described by Buenrostro et al. (2013) and indexed using a TruePrep DNA Library Prep Kit (TD501, Vazyme, Nanjing, China). Approximately 50,000 cells in each biological replicate were harvested and dissociated using a cell strainer via centrifugation (750 × *g* for 5 min) at room temperature. The cell pellets were resuspended in lysis buffer (10 mM Tris-HCl, pH 7.5, 10 mM NaCl, 3 mM MgCl_2_, 0.1% NP-40), then centrifuged at 500 × *g* for 15 min at 4°C. The supernatant was discarded. The pelleted nuclei were immediately submitted to tagmentation reaction using Tn5 transposase (TTE Mix V50) for 30 min at 37°C. DNA purified using the Qiagen PCR purification MinElute Kit (Qiagen, Valencia, CA, USA) was eluted in 10 μl of elusion buffer, indexed, and amplified. All libraries were cleaned using VAHTS DNA Clean Beads and sequenced on the Illumina Novaseq 6000 (Illumina, provided by Annoroad Genomics, China). Three independent biological replicates were performed for each mDPC-D0 and D9, whereas two independent biological replicates were performed for each time point in the time-course ATAC-seq. However, one replicate was performed for OC-positive and OC-negative cells ATAC-seq.

### Cleavage under targets and tagmentation library preparation

We cultured mDPCs in a mineralization medium and normal culture medium for 0 and 9 days. Cleavage under targets and tagmentation (CUT and Tag) libraries were prepared as previously described by Kaya-Okur et al. (2019) and indexed using an *In-Situ* ChIP Library Prep Kit (TD902, Vazyme, Nanjing, China). Approximately 10,000 mDPCs in each biological replicate were harvested and centrifuged (600 × g) at room temperature for 3 min. The supernatant was washed and resuspended in Wash Buffer supplemented with protease inhibitors (Roche Complete Protease Inhibitor EDTA-Free Tablet, Sigma-Aldrich, St. Louis, MO, USA). Concanavalin A-coated magnetic beads in each sample were washed and resuspended in binding buffer. Then beads were added to the cells, gently vortexed, and incubated in a shaker for 10 min at room temperature. The unbound supernatant was removed. The bead-bound cells were resuspended in precooling antibody buffer [2 mM EDTA, 0.1% BSA in DIG Wash Buffer (0.05% digitonin in wash buffer)] and incubated with (1:50 dilution) primary antibody against H3K27ac (ab4729, Abcam, Cambridge, MA, USA) in a shaker overnight at 4°C. The primary antibody on the magnet stand was removed, and then a secondary antibody (Guinea Pig Anti-Rabbit IgG antibody, 611-201-122, Rockland Immunochemicals, PA, USA) was diluted (1:100) in DIG Wash buffer and incubated with cells at room temperature for 1 h. Cells were washed in DIG Wash buffer using the magnet stand to remove unbound antibodies. A dilution of hyperactive pA-Tn5 transposon complex (0.04 µM) was prepared in DIG-300 buffer supplemented with 0.01% digitonin and protease inhibitors. Then cells were incubated with pA-Tn5 transposon complex in a shaker at room temperature for 1 h. Subsequently, cells were resuspended in tagmentation buffer (10 mM MgCl_2_ in DIG-300 buffer) and incubated at 37°C for 1 h. To terminate tagmentation, we added 10 µl of 0.5 M EDTA, 3 µl of 10% SDS, and 2.5 µl of 20 mg/ml proteinase K to each sample, followed by overnight incubation at 37°C. Purified DNA was amplified and indexed. The libraries were cleaned using VAHTS DNA Clean Beads (N411, Vazyme, Nanjing, China) and sequenced on the Illumina Novaseq 6000 (Illumina, provided by Annoroad Genomics, China). We applied 150-bp pair-end sequencing with a sequencing depth of 6G base pair raw data (generated approximately 20 million mapped paired reads). Three independent biological replicates were performed for each mDPC-D0 and D9.

### Cleavage under targets and tagmentation and Transposase-accessible chromatin assay with high-throughput sequencing library analyses

Raw reads of CUT and Tag and ATAC-seq were first subjected to trimmomatic (v.0.38) (Bolger et al., 2014) for adaptor trimming. We did a quality check using FastQC (https://www.bioinformatics.babraham.ac.uk/projects/fastqc/) before alignment to ensure proportionate quality libraries. Then the paired-end sequencing reads were aligned to the mouse genome (mm10) using Bowtie 2 (Langmead and Salzberg, 2012). SAMtools (Li et al., 2009) was applied to eliminate the PCR duplicates. DeepTools2 (Ramírez et al., 2016) was used to generate bigwig files. MACS2 (v.2.1.1) (Zhang et al., 2008) was applied for peak calling. Comparison of differentially accessible NFRs between different time points was achieved using DiffBind (DESeq2 v.1.26.0) (Love et al., 2014). We further applied the Homer package (Heinz et al., 2010) to identify the *de novo* motifs enriched in the NFRs of different mineralization time points. Genomic Regions Enrichment of Annotations Tool (GREAT) (McLean et al., 2010) was adopted to annotate differentially accessible NFRs and perform GO enrichment assay. Eventually, coverage plots for CUT and Tag and ATAC-seq results were generated using DeepTools2 and uploaded to the UCSC genome browser. All correlative graphs were plotted using R scripts in RStudio (v.February 1, 5001). For footprint, mapped reads at the groups were concatenated and subjected to CENTIPEDE (Pique-Regi et al., 2011), and cutting frequency near Dmp1 3′UTR was then visualized. Example scripts were uploaded to Github (https://github.com/Badgerliu/mDPC_epi_paper). All scripts are available upon request.

### Integration of transposase-accessible chromatin assay with high-throughput sequencing and H3K27Ac cleavage under targets and tagmentation data

To identify odontoblast stage-specific active enhancers, we integrated H3K27Ac CUT and Tag peaks to ATAC-seq at the same time point following our previously published strategy (Liu et al., 2020). The regions flanked by two adjacent H3K27Ac peaks less than 1,500 bp were defined as “H3K27Ac-flanked” regions. D0- or D9-enriched NFRs were “intersected” with D0- or D9-enriched H3K27Ac peaks or “H3K27Ac flanked” regions using bedtools. The overlapped NFRs were termed as D0- or D9-enriched active enhancers.

### Immunohistochemistry

The isolated mandibles of wild-type C57BL/6 mouse at PN2 (for anti-ATF5) and OC-Cre, and Rosa26-mTmG (for OC-positive lineage tracing) were fixed in 4% paraformaldehyde at 4°C for 24 h. The samples were then treated in 10% ethylenediaminetetraacetic acid (EDTA) for 1–2 days, dehydrated, and embedded in paraffin. Sagittal sections (5-µm thick) were dewaxed and rehydrated. After being boiled in 1 mM citrate buffer (pH = 6.0) for 15 min, slides were cooled down to room temperature. Subsequently, histological sections were blocked in bovine serum albumin (BSA) (Biosharp, China) and incubated with antibodies against GFP (1:100, Cat. No. ab13970, Lot No. GR89472–15; Abcam, MA, USA) or ATF5 (1:100; Cat. No.ab184923, Lot No. GR282324-13; Abcam, MA, USA) at 4°C overnight. For regular immune chemical color reaction, samples were reacted with polymer Helper and poly-HRP-anti-rabbit IgG or poly-HRP-anti-goat IgG at room temperature for 15 min after they were washed with phosphate-buffered saline (PBS). The samples were detected using a diaminobenzidine (DAB) reagent kit (Maixin) and counterstained with hematoxylin. For immunofluorescent analysis, we performed anti-GFP staining in Alexa 633 (anti-Chicken lgY, 1:500, Cat. No. A21103, Lot No. 2079359; Thermo Fisher Scientific, MA, USA) and counterstained with DAPI (Life Technologies, USA). Pseudocolor was performed using Fiji (Schindelin et al., 2012) to convert color of Alexa633 to GFP.

### Quantitative reverse transcriptase PCR analysis

Total RNA from cells was extracted using HP Total RNA Kit (Omega biotech, Norcross, GA, USA), then reverse transcribed into cDNA using Reverse Transcription System (Life Technologies). qPCR was performed in CFX Connect Real-Time PCR system (Bio-RAD) using ChamQ SYBR qPCR Master Mix (Vazyme). Gapdh, Atf5, Alp, and Dmp1 were quantified with Gapdh as the internal normalization control. The RNA expression ratio was denoted as “mean ± standard deviation” from three independent biological replicates.

### Western blot analysis

After different treatments, mDPCs were lysed in lysis buffer (Feiyi Technology, China) and centrifuged at 13,000 rpm for 10 min at 4°C. Total protein was quantified. After that, Western blot was performed with the following antibodies.

DMP1 (1:1,000; Cat. No. ab103203, Lot No. GR3212251-2; Abcam, MA, USA), DSP (1:1,000; Cat. No. NBP1-91612, Lot No. QC6694; NOVUSBIO, CO, USA), ATF5 (1:2,000; Cat. No. ab184923, Lot No. GR282324-13; Abcam, MA, USA), and β-ACTIN (1:8,000; BioPM, Beijing, China).

### Statistical analysis

All results were presented as “mean ± standard deviation (SD).” One-way ANOVA was performed for multiple group comparisons, whereas a two-tailed *t*-test was performed for two groups. Values of p < 0.05 were considered statistically significant.

## Results

*In vitro* odontoblastic differentiation resembles crown odontoblast differentiation trajectory of OC-positive odontogenic cells.

**FIGURE 1:**
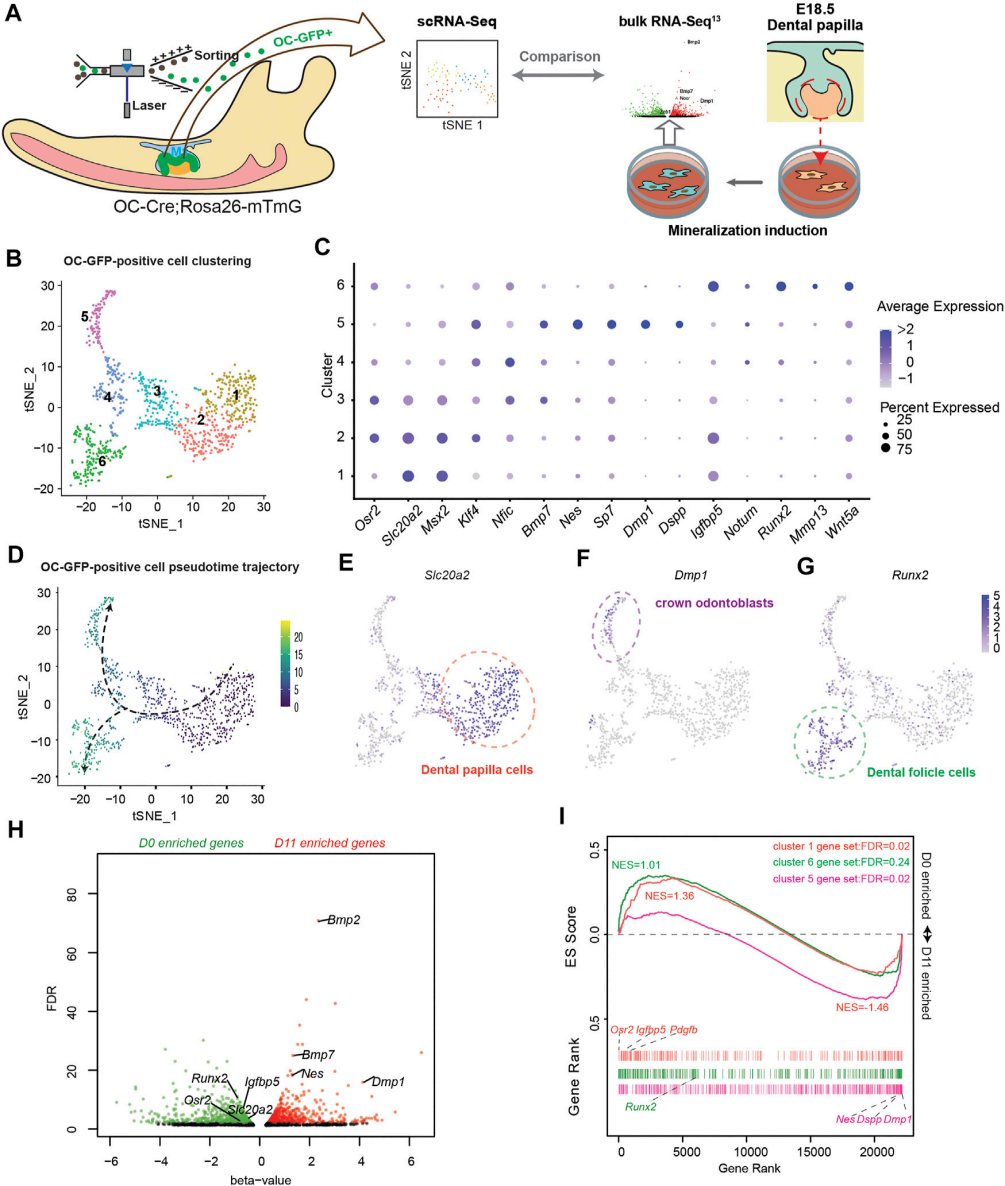
Comparison between *in vitro* odontoblastic differentiation and *in vivo* osteocalcin (OC)-positive odontogenic lineage differentiation. **(A)** Study design comparing single-cell RNA-seq (scRNA-seq) using OC + odontogenic lineage from postnatal day 0 (PN0) first lower molar tooth germ and bulk RNA-seq of dental papilla cells before and after mineralization induction. **(B)** *t*-distributed stochastic neighbor embedding (tSNE) depicting the clustering of 975 single-cell transcriptional profiles obtained from FACS-sorted GFP-positive cells from OC-Cre and Rosa26-mTmG PN0 first lower molar tooth germ. **(C)** Dot-plot showing expression and enrichment of selected top genes identified in each cluster. The size of dot indicate the percentage of cells per cluster. **(D)** Pseudotime-trajectory of OC-GFP-positive scRNA-seq. **(E)** *Slc20a2* marks pre-odontoblast population in OC + odontogenic lineage. **(F)** *Dmp1* marks crown odontoblast population in OC + odontogenic lineage. **(G)** *Runx2* marks dental follicle cell population in OC + odontogenic lineage. **(H)** Volcano scatter plot showing the differentially expressed genes revealed in bulk RNA-seq from dental papilla cells before (D0) and after (D11) mineralization induction. Red dots indicate genes significantly enriched in D11, and green dots indicate genes significantly enriched in D0. **(I)** Gene set enrichment analysis (GSEA) using marker genes from cluster 1 (orange), cluster 6 (green), and cluster 5 (violet) as gene sets comparing expression profiles from D0 and D11 bulk-RNA-seq. NES, normalized enrichment score; FDR, false discovery rate, as generated in GSEA.

Previous studies on odontoblast differentiation mainly employed an *in vitro* model using mouse dental papilla cells (mDPCs) induced by a mineralization medium. They reported significant upregulation of *Dmp1*, *Dspp* (Chen et al., 2009), and *Nestin* (*Nes*) (Kaukua et al., 2014), which are marker genes mainly expressed in the odontoblast layer [*Nes* is also expressed in the pericytes in dental pulp (Yianni and Sharpe, 2018)]. However, the same medium could also potentially induce osteogenic differentiation when bone marrow-derived mesenchymal cells (BMSCs) are used (Yu et al., 2021). Thus, whether this model can mimic *in vivo* odontoblast differentiation remains unclear. To clarify this, we first attempted to compare the transcriptomes of *in vitro* with *in vivo* odontoblast differentiation models (Figure 1A). Inspired by the specific Cre activity in the odontoblast layer of OC-Cre mice (Yun et al., 2016), we generated OC-Cre and Rosa26-mTmG mice to assess odontoblast differentiation *in vivo*. GFP activity was mainly localized at the odontoblast layer (Supplementary Figures 2A, D) and dental follicle cells in developing tooth root at postnatal day 0 (PN0) in the first lower molar. Also, some blood vessels were GFP positive (Supplementary Figure 2C). We isolated GFP-positive cells from PN0 first lower molar tooth germ of OC-Cre and Rosa26-mTmG mice, and performed single-cell RNA-seq. Considering the initial cluster and expression of *Dmp1* and Cd34, we isolated 975 cells associated with *Dmp1*, which was termed as “OC-positive odontogenic lineage.” Six clusters were revealed (Figure 1B). Following the assessment of the significant differential marker genes (minimal percentage as 25% and *p* < 0.01; Supplementary Table 1), the cell distribution was revealed to resemble a subset of population single-cell RNA-seq reported from the apical halves of molar at PN7.5 (Wen et al., 2020). Clusters 1 and 2 were identified as dental papilla cells, expressing high levels of *Slc20a2*, *Osr2*, and *Dlx3* (Figures 1C, E; Supplementary Figures 3, 4) (Yamashiro et al., 2003). Cluster 5 was termed as odontoblast with explicitly high expression of *Dmp1*, *Nes*, and *Dspp* (Figures 1C, F; Supplementary Figures 3 and 4). *Runx2*, *Mmp13*, and *Bmp3* were highly enriched in cluster 6 (Figures 1C, G; Supplementary Figures 3 and 4); they were previously identified to be highly associated with dental follicle cells (Wen et al., 2020). Clusters 3 and 4 were regarded as transient status from OC-positive dental papilla cells to odontoblasts or dental follicle cells; they exhibited gradually low expression of *Slc20a2* and *Msx2*. Differentiation trajectory of these OC-positive odontogenic lineages based on gene expression changes was inferred (Figure 1D). Dental papilla cells (clusters 1 and 2) were the start point, whereas two destinations were crown odontoblasts (cluster 5) and dental follicle cells (cluster 6). These results demonstrated that OC-positive odontogenic lineage contributed to crown odontoblasts and dental follicle cells.

To make a transcriptome-wide comparison between *in vivo* and *in vitro* odontoblast differentiation, we adopted our previously published bulk RNA-seq profile using mDPCs treated with mineralization medium for 0 days (D0) and 11 days (D11) (Lin et al., 2021) (Figure 1H). *Zeb1* (Xiao et al., 2021a) and *Sall1* (Lin et al., 2021) were upregulated on day 11 and promoted odontoblastic differentiation. Also, the enrichment of *Osr2*, *Slc20a2*, and *Runx2* on D0 was not found in the crown odontoblast population (cluster 5) in the scRNA-seq profile. We applied gene set enrichment analysis (GSEA) with marker genes in clusters 1, 5, or 6 as three independent gene sets and found that genes enriched on D11 were positively correlated with cluster 5 gene set (NES = −1.46, FDR = 0.02, no. of permutations = 10,000), whereas D0 was positively correlated with cluster 1 gene set (NES = 1.36, FDR = 0.02). However, genes in the cluster 6 set exhibited an even distribution in both D0 and D11 (NES = 1.01) (Figure 1I) indicating it was not like the *in vitro* differentiation. These findings suggested that *in vitro* odontoblast differentiation using mDPCs from E18.5 concurs with *in vivo* odontoblast differentiation of OC-positive odontogenic lineage.

### Identification of odontoblast-specific active enhancers

**FIGURE 2:**
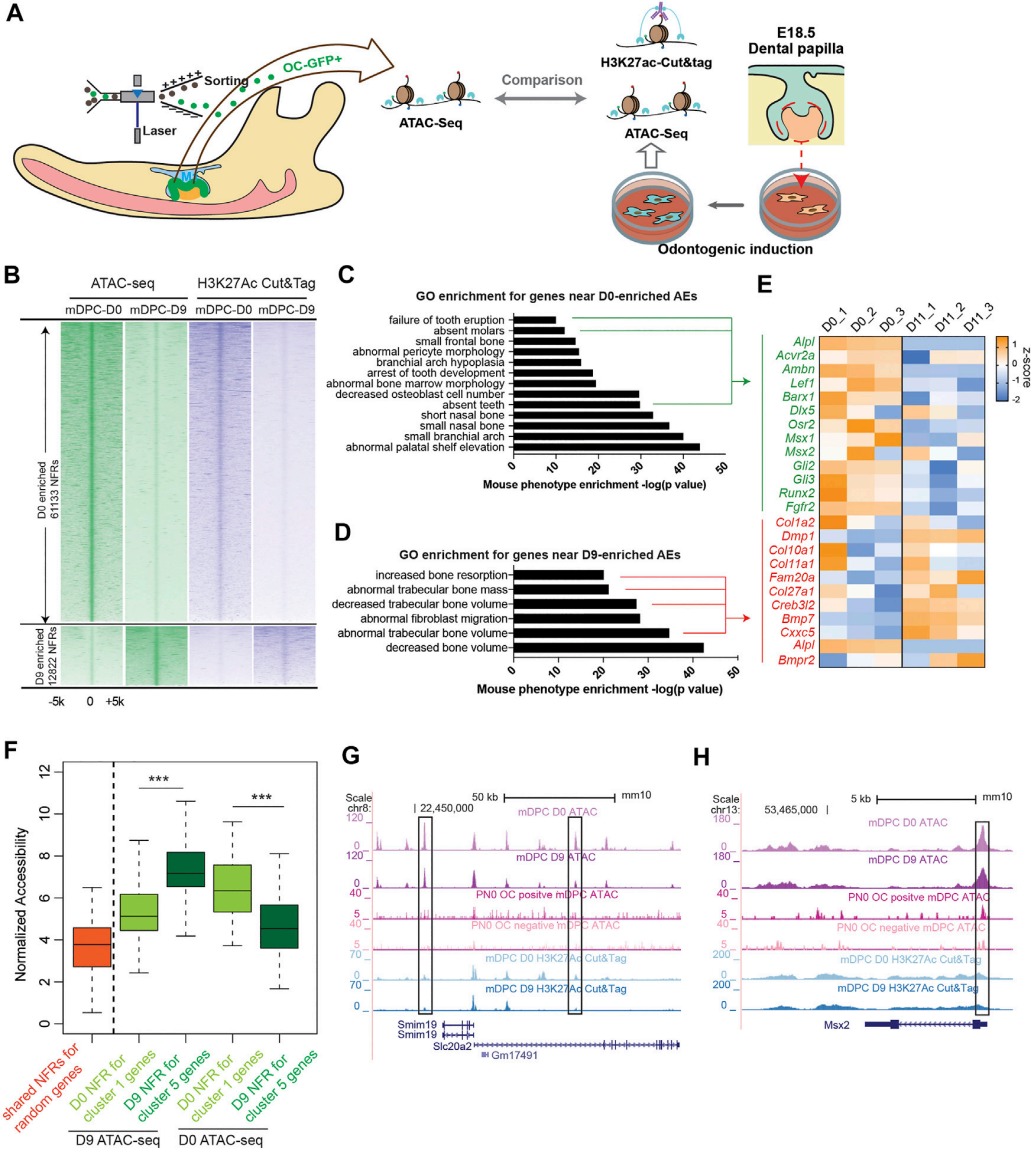
Transposase-accessible chromatin assay with high-throughput sequencingATAC-seq) and H3K27Ac cleavage under targets and tagmentation (CUT and Tag) from both *in vitro* and *in vivo* odontoblast differentiation revealed chromatin early shaping predetermined dentinogenesis potential of dental papilla cells. **(A)** Study design comparing ATAC-seq from *in vitro* and *in vivo* odontoblast differentiation and annotation for active enhancers using H3K27Ac CUT and Tag. **(B)** Density plot of aligned ATAC-seq and H3K27Ac CUT and Tag peaks differentially enriched in D0 and D9 during mineralization of E18.5 dental papilla cells. Each line is centered on the nucleosome-free region (NFR) with significantly more reads in D0 or D9 ATAC-seq. Reads in H3K27Ac CUT and Tag are aligned to the same NFR. Gene ontology enrichment using “Mouse Phenotype Single KO” for D0- **(C)** and D9- **(D)** enriched active enhancers (AEs). **(E)** Expression changes of the overlapped genes from bulk RNA-seq profile in indicated GO terms visualized in heatmap. **(F)** Plot of accessibility scores (generated in DiffBind) of elements with differential accessibility associated with genes differentially expressed in cluster 1 and cluster 5, showing elements with increased accessibility in D9-*in-vitro*-differentiated odontoblast tend to be associated with genes enriched in cluster 5, and vice versa. ***p < 0.001, by Kolmogorov–Smirnov test. **(G, H)** UCSC genome browser view showing ATAC-seq and CUT and Tag peaks near the *Slc20a2* and *Msx2* loci.

Activation and deactivation of *cis*-regulatory elements have been directly associated with transcriptional regulation (Heinz et al., 2010). We explored whether there are such elements that regulate transcription during odontoblast differentiation. The above *in vitro* and *in vivo* models were employed for ATAC-seq to identify open chromatin regions before and after odontoblast differentiation. The open chromatin regions in the *in vitro* models were annotated using H3K27Ac CUT and Tag to reveal active enhancers (Figure 2A). We found 27,484 and 26,215 nucleosome-free regions (NFRs) enriched in OC-positive and OC-negative cells (Figure 2B). We compared the ATAC-seq reads from *in vitro* odontoblast differentiation and found no significant difference between D0 and D11 ATAC-seq in the OC-positive enriched NFRs. However, the overall D0 and D11 ATAC-seq signals in the OC-positive enriched NFRs were significantly higher than those in OC-negative ones (*p* < 0.001, by Kolmogorov Smirnov test, *ks*-test) (Supplementary Figure 6A). Considering the transcriptome comparison between *in vitro* and *in vivo* differentiation, this is reasonable given that OC-positive odontogenic lineage covers the transition from D0 to D11. Besides, the shift in chromatin accessibility from D0 to D11 reflected detailed changes of *cis*-regulatory elements during odontoblast terminal differentiation. We, therefore, chose this *in vitro* model for further analysis.

As for the ATAC-seq from *in vitro* odontoblast differentiation model, there were 61,133 NFRs enriched in D0 (before mineralization induction) and 12,822 enriched in D9 [log_2_ (fold change) >0.5 or <0.5, FDR <0.01, by DESEQ2; Supplementary Tables 2 and 3]. The enrichment of ATAC-seq reads correlated with H3K27Ac CUT and Tag (Figure 2B and Supplementary Figures 7A, B). The NFRs overlapped or flanked by H3K27Ac signals at the same stage were defined as active enhancers (AEs); also, we found 12,221 AEs on D0 and 2,461 on D9 (Supplementary Figure 7C and Tables 4 and 5). In several regions near Dmp1 locus (Supplementary Figure 7D), the accessibility remained unchanged or decreased post odontoblast differentiation. However, the H3K27Ac CUT and Tag signals were significantly elevated. This is common during the establishment of enhancer activity: H3K27Ac modification follows the “opening” of chromatin regions (Calo and Wysocka, 2013). We analyzed the GO enrichment for the associated genes of D0- and D9-enriched NFRs in “Mouse phenotype single KO.” Genes associated with D0-enriched NFRs were more enriched in odontogenic-related GO terms such as “failure of tooth eruption” and “absent teeth” (Figure 2C), including *Acvr2a*, *Lef1*, and *Msx1*. The expressions of these genes were low in D11 according to the RNA-seq profile. Transient transfection-based dual-luciferase assay in mDPCs confirmed elements near *Msx1* (*Msx1*-0.6; mm10 chr5: 37,824,275–37,824,941) and *Fgfr2* (Fgfr2+96; mm10 chr7: 130,167,270–130,167,658; and Fgfr2-34; chr7: 130,298,850–130,299,791) exhibited higher enhancer activity on D0 compared with D11, despite the possible loss of plasmids during odontoblast differentiation (Supplementary Figure 8). Moreover, genes associated with D9-enriched NFRs were highly associated with mineralization GO terms, such as “increased bone resorption” and “decreased trabecular bone volume/mass” (Figure 2D). RNA-seq profile demonstrated that the genes, *Dmp1* and *Creb3l2*, were also significantly upregulated on D11 (Figure 2E). We performed *in vitro* validation and found that two elements targeting these genes (Col1a2-8.3; chr6: 4,497,230–4,497,895; and Bmp7+67; chr2: 172,872,129–172,872,743) exhibited high enhancer activity (Supplementary Figure 9).

We also found that differentially accessible NFRs associated with cluster 5 genes (in scRNA-seq) had significantly higher average accessibility from mDPCs D9 than those from mDPCs D0, and vice versa (Figure 2F). Particularly, Msx2 and Slc20a2 were highly expressed in cluster 1, and there were more intense signals of ATAC-seq along with H3K27Ac in the mDPC D0 group (Figures 2G,H). Collectively, this analysis suggested that chromatin accessibility was associated with the terminal differentiation of odontoblast.

Time-course ATAC-seq reveals that dynamic changes in open chromatin regions predetermine terminal differentiation of odontoblast.

**FIGURE 3:**
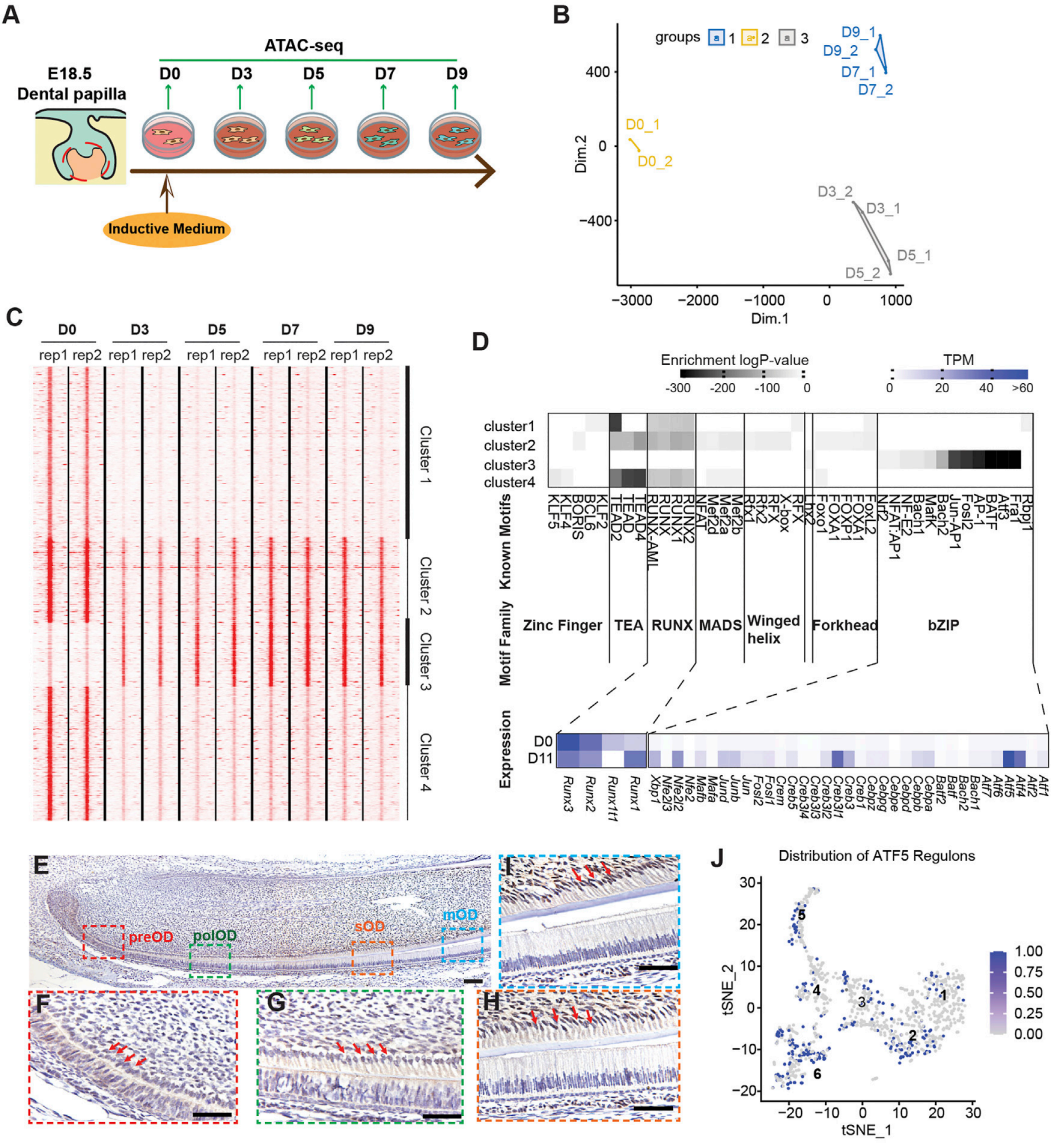
Time–course ATAC-seq integrated with RNA-seq identified transcription factors (TFs) enriched in open chromatin regions associated with odontoblast terminal differentiation. **(A)** Study design for time-course ATAC-seq using *in vitro* odontoblast differentiation. **(B)** A multidimensional scaling (MDS) plot showing the difference between differential-enriched NFRs among D0, D3, D5, D7, and D9 ATAC-seq. **(C)** k-mean clustering of differential-enriched NFRs across replicates and stages, highlighting peak density profile. Each line is centered in NFRs enriched in different stages, with expansion from −5,000 to +5,000 bp. **(D)** Top enriched known motifs in each cluster as predicted via Homer analysis. The color gradient indicates the log_10_ (*p*-value) of enrichment analysis over the total background peaks using binomial testing. Hierarchy clustering of TF distribution based on log_10_ (*p*-value) was depicted in the heatmap. Coincidently, most of the clustered TFs belong to a specific TF family with a similar structure, such as zinc-finger, RUNX, forkhead, or bZIP. Of note, the bZIP TFs family was specifically enriched in cluster 3-enriched NFRs. The average expression of each member in the bZIP family on D0 and D11 bulk RNA-seq profiles was exhibited in the heatmap. **(E–I)** Immunohistochemistry of the expression pattern of ATF5 in PN2 murine lower incisor. ATF5 expression in the pre-odontoblasts (preOD) **(F)**, polarized odontoblasts (polOD) **(G)**, secretory odontoblast (sOD) **(H)**, and mature odontoblast (mOD) **(I)** were gradually increasing. Scale bar: 100 µm. Red arrows point to the odontoblast layer. **(J)** tSNE plot showing the distribution of cells with abundant ATF5-targeting genes (regulons) in OC-positive scRNA-seq profile.

Because NFRs enriched in mDPC-D9 groups are associated with odontoblast terminal differentiation, we explored whether these regions were regulated by any specific TFs. We performed a more detailed time-course ATAC-seq during *in vitro* odontoblast differentiation at 2-day intervals (Figure 3A). Following the analysis of differentially enriched NFRS in each group, we found that the accessibility of NFRs changed dramatically from D0 to D3 (Figure 3B), much earlier than the upregulation of odontoblast-specific genes, *Dmp1* and *Dspp*, which are always, in most cases, increased around day 5 or day 7 post-induction (Tao et al., 2019). There were 5,526 NFRs differentially enriched between the D0 and D3 group, 48 between D3 and D5, 131 between D5 and D7, and no different NFRs between D7 and D9 (Supplementary Figure 10). High-resolution analysis across all the merged consensus peaks from D0 to D9 using k-means clustering revealed four clusters of elements with two major trends of chromatin accessibility, with the NFRs in clusters 1, 2, and 4 gradually “closed”, and cluster 3 gradually “open” (Figure 3C). The promoter of *Gli1* (Supplementary Figure 11A) and an element near *Klf4* (Supplementary Figure 11B) gradually lost accessibility during differentiation. GO enrichment assay for NFRs revealed that genes near cluster 1 (including *Gli1*) were associated with “incisor/tooth morphology” indicating their odontogenic function, whereas genes near cluster 3 were associated with “bone mass” (Supplementary Figure 12). These underpinned cluster 3 are the very NFRs associated with odontoblast terminal differentiation, given by mineralization as the major biological process.

We applied Homer to identify the known TFs motifs enriched in the four clusters, which allowed us to find the specific TFs that regulate NFR activity. We found a well-separated pattern of the TF family in the four clusters of NFRs. Zinc Finger, TEA, RUNX, MADS, and Forkhead TF families were exclusively enriched in clusters 1, 2, and 4, whereas the basic region/leucine zipper (bZIP) family members were enriched in cluster 3. To identify the possible missing TF members in the bZIP family attributed to the incomplete database, we examined the changes in expression level and abundances of all the recorded bZIP TF (from UniProtKB database) (UniProt, 2019) referring to our bulk RNA-seq data. Most of the bZIP family members detected in bulk RNA-seq were upregulated post odontoblast differentiation. In contrast, RUNX family members, enriched only in clusters 1, 2, and 4 were downregulated (Figure 3D). Of the bZIP family members, we selected *Atf5* for further validation. IHC results revealed that ATF5 was highly expressed in the secretary odontoblasts and mature odontoblasts, but weakly expressed in the pre-odontoblast in the odontoblast layer of the lower incisor from PN0 mouse (Figures 3E–H and Supplementary Figure 14). Additionally, SECNIC imputation demonstrated that the regulons of ATF5 in the scRNA-seq profile were mainly distributed in clusters 2, 3, 4, 5, and 6, which depicted its positive role in the differentiation of odontoblast and dental follicle cells (Figure 3J). Furthermore, we scanned all the target NFRs of ATF5 motifs in cluster 3 and found that the genes associated with these NFRs mediated “ossification” and “bone development” (Supplementary Figure 13). Taken together, our time-course ATAC-seq experiments with motif analyses revealed that the dynamic changes in chromatin accessibility occur earlier than the transcriptional changes and predetermined odontoblast-related gene transcription.

### ATF5 promotes odontoblastic differentiation partially by binding to a Dmp1 enhancer

**FIGURE 4:**
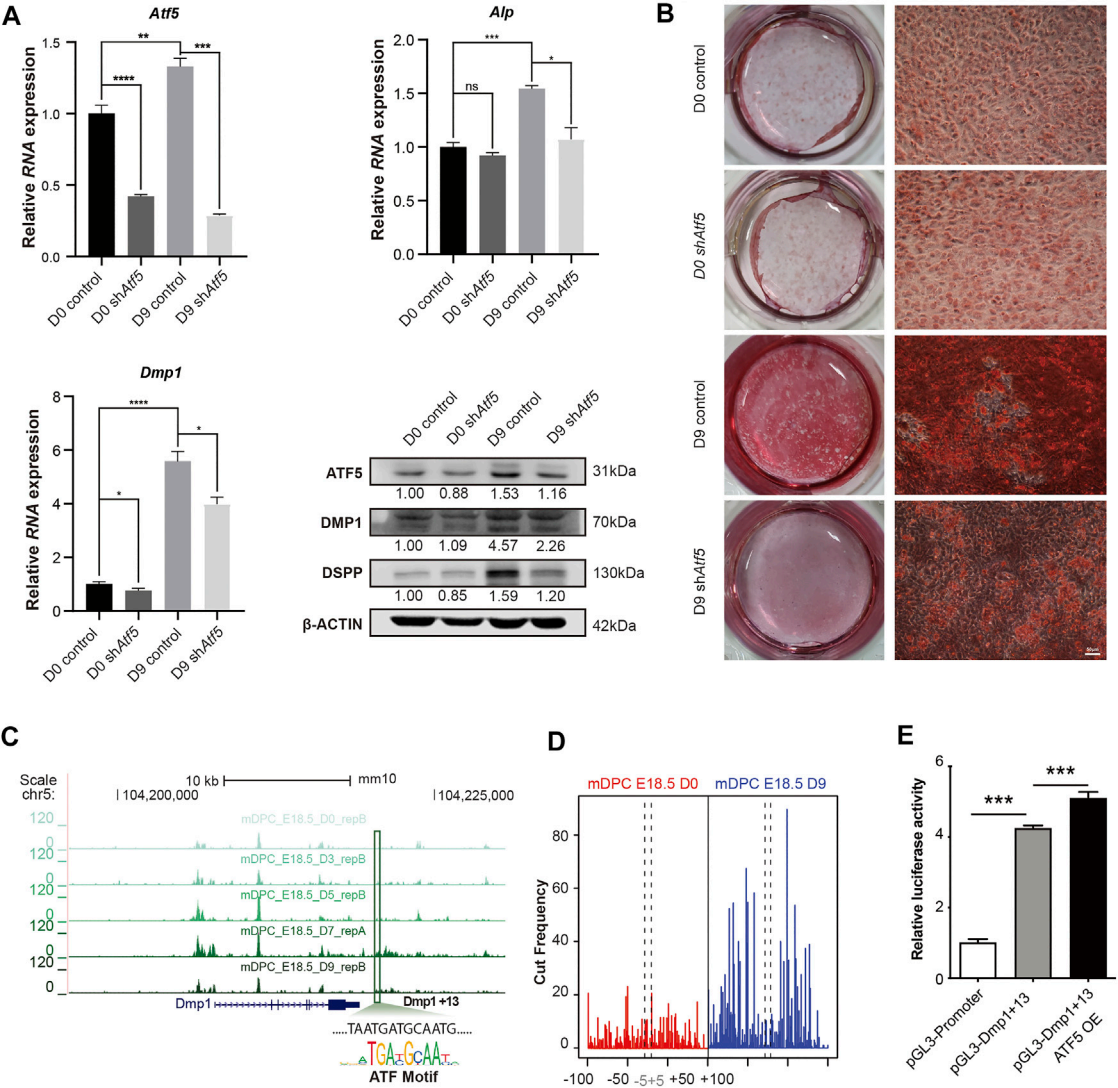
ATF5 promotes odontoblast terminal differentiation by activating a *Dmp1*-related enhancer. Primary mDPCs of E18.5 mice cultured in mineralization induction medium for 0 days (D0) and 9 days (D9) after infection with lentivirus-expressing shRNA against Atf5 and empty control. **(A)** qRT-PCR and Western blot showing expression of related odontoblast markers in different conditions. **(B)** Alizarin red staining showing fewer mineralization nodules on D9 in sh*Atf5* group compared with control. **(C)** A chromatin region in the downstream of *Dmp1* (Dmp1+13) gradually gained accessibility (belonged to cluster 3 in Figure 3) in time–course ATAC-seq with a typical binding motif for the ATF family. **(D)** Tn5-transposase footprint based on CENTIPEDE revealed the ATF-binding motif in **(C)** “protected” on D9 ATAC-seq. **(E)** Dual-luciferase assay showing the enhancer activity of Dmp1+13 could be promoted by the overexpression of ATF5 in mDPC cells. *p < 0.05, **p < 0.01, ***p < 0.001.

We further characterized the function of ATF5 in the *in vitro* odontoblast differentiation model. We applied lentivirus-mediated shAtf5 transduction with scrambled plasmid as a control. qPCR and Western blot demonstrated that *Atf5* knockdown significantly reduced RNA levels of *Dmp1* and *Alp* on day-9 post induction. A significantly lower protein level of DSPP was reported in the sh*Atf5* group on day 9 (Figure 4A and Supplementary Figure 15). Alizarin red results demonstrated that mineralization was significantly inhibited in the *Atf5*-knockdown group (Figure 4B). Based on our previous analysis of the target genes of the ATF5 motif in cluster 3 NFRs, Dmp1 was selected as a target of ATF5 for further exploration. We found an element in the downstream of *Dmp1* (Dmp1+13; chr5: 104,216,600–104,216,700) with ATF5 motif (Figure 4C), accessibility of which was significantly increased. CENTIPEDE-based Tn5 transposase footprinting assay (Pique-Regi et al., 2011) was performed using two concatenated replicates from mDPC D0 and D9 ATAC-seq. Despite the fact that there were more mapped reads in D0 than D9, footprint revealed a higher cut frequency of Tn5 flanking this motif on D9 mDPC ATAC-seq (Figure 4D), an implication that this site was protected by protein. Furthermore, ATF5 overexpression significantly increased the enhancer activity of Dmp1+13 (Figure 4E). Collectively, these findings affirmed that ATF5, a major TF enriched in odontoblast-related NFRs, potentially promoted odontoblast terminal differentiation partially via the induction of *Dmp1*-related enhancer activity.

## Discussion

**FIGURE 5:**
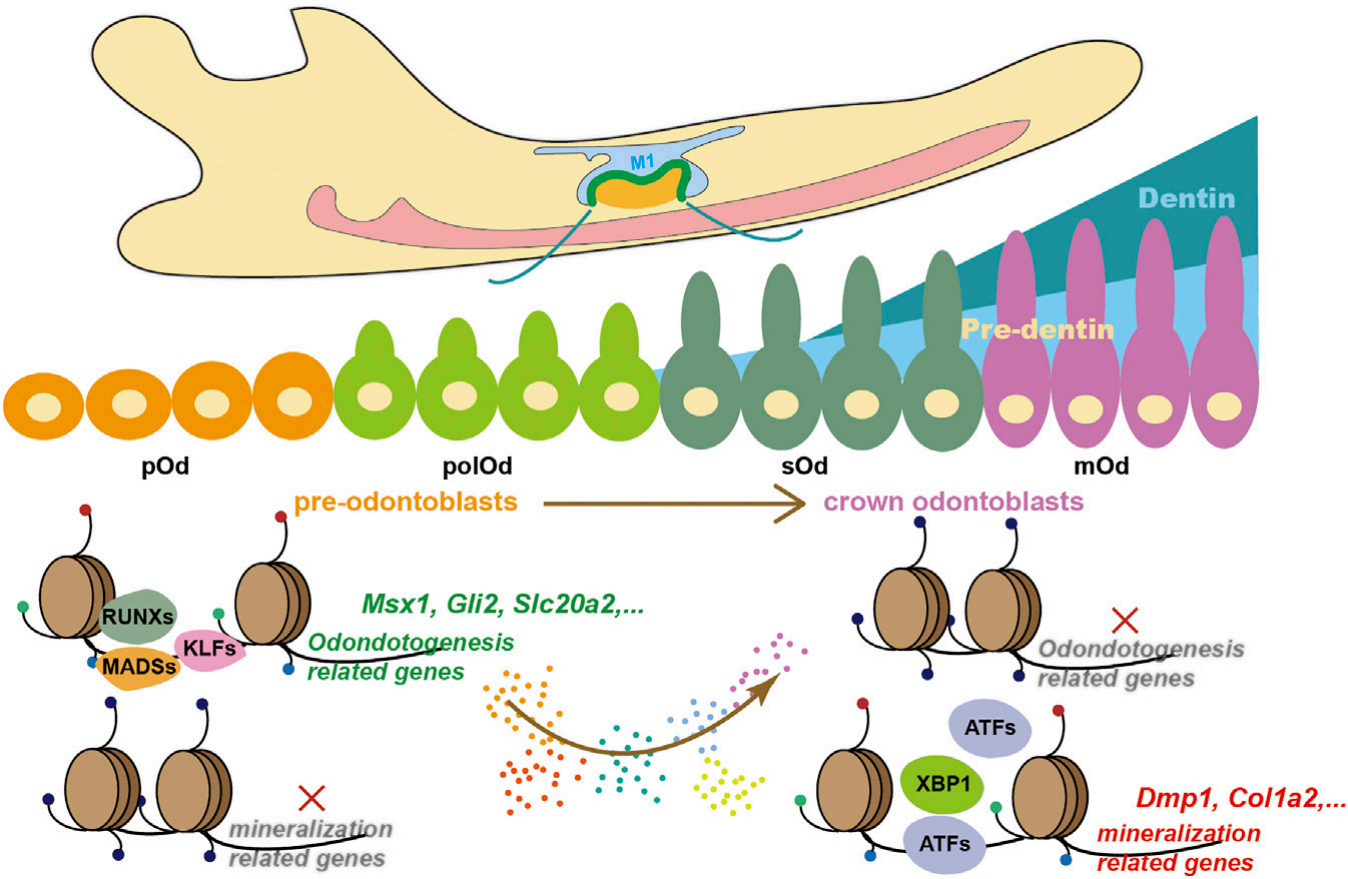
A proposed working model describing how chromatin accessibility predetermines odontoblast terminal differentiation.

The mechanism by which TFs interact with chromatin to regulate gene expression and influence cell fates is one of the leading questions in the genome and developmental biology. A previous study combining *in vivo* genetic lineage tracing and bulk RNA-seq along with ChIP-seq against histone modifications in postnatal dental pulp perivascular-derived mesenchymal stem cells illustrated that odontoblast-specific genes, such as *Dspp* and Dmp1, were in a transcriptionally permissive state inhibiting by RING1B (Yianni and Sharpe, 2018). However, during embryonic development, how the fate of odontoblast lineage is initiated remained unclear. The variation in regional accessibility is the first step for chromatin landscape alternation (Calo and Wysocka, 2013). In this study, we described the transcriptome changes during odontoblast terminal differentiation via single-cell RNA-seq from OC-positive odontogenic lineage combined with bulk RNA-seq from mDPC induced by mineralization medium. Following the identification of marker genes, we found that the increase in chromatin accessibility of these markers, such as *Dmp1* (Balic and Mina, 2011) and *Dspp* (Chen et al., 2004), occurred much earlier than the initiation of transcriptional upregulation. This integrated analysis underpinned the predecisive roles of chromatin accessibility during odontoblast terminal differentiation (Figure 5).

At the end of differentiation, marker genes upregulated along with the enriched NFRs were associated with the major biological process of odontoblast, including GO terms such as “bone mass” and “bone volume.” Because several odontoblast markers and the same induction medium are shared with osteoblast, it is unclear whether the *in vitro* induction is via odontoblast or osteoblast differentiation. *Dmp1* and *Dspp* are two marker genes for odontoblast differentiation; however, loss of either of them causes bone defect (Verdelis et al., 2008; Sun et al., 2010). Our integrated analysis between single-cell RNA-seq from odontogenic lineage and bulk RNA-seq from *in vitro* odontoblast induction revealed that mineralization using mDPC isolated from E18.5 lower molar is comparable with crown odontoblast differentiation at the transcriptional level. *Runx2* is a positive regulator of osteoblast differentiation but is nearly undetectable after the bell stage of a tooth (Chen et al., 2009) and was significantly down-regulated during *in vitro* differentiation. Also, NFRs with *Runx2* gradually lost accessibility during this biological process. These results imply that “downregulation of *Runx2*” may be a crucial aspect to differentiate between odontoblast and osteoblast differentiation. We also noticed that there were more open chromatin regions in the odontoblast *in vitro* at D0 than OC-positive and -negative cells. This may be due to the batch effects in sequencing and culture condition. Nevertheless, the *in vivo* model is the best validation method for whether a gene potentially promotes odontoblast differentiation.

Other than terminal differentiation, we revealed that NFRs near odontogenic genes, including *Gli2*, *Msx1*, and *Runx2*, were gradually “closed.” These genes have been extensively explored for their roles in early odontogenesis (Alappat et al., 2003), and any defect would lead to hereditary tooth abnormality (Hardcastle et al., 1999) or tooth agenesis (Mundlos et al., 1997). The loss of these odontogenic potential may be attributed by the long-term *in vitro* culture or the differentiation, whereby the differentiation of dental papilla cells becomes irreversible. Compelling evidence shows that dental mesenchymal cells (Yoshikawa and Kollar, 1981) or dental pulp stem cells (Hu et al., 2014) after PN0 cannot form tooth when recombined with dental epithelium before E11.5. In our recent work, we analyzed the role of Zeb1 and Sall1 in the regulation of odontoblast lineage and found that these 2 TFs can only alter odontogenic-related NFRs in mDPCs from E16.5 but not PN0 lower molars. These results suggested that despite the effect of culture, mDPCs at late embryonic stages (i.e., PN0) lose their odontogenic potential as depicted by the loss of accessibility of chromatin regions.

Assessment of transcription factor motifs enriched in ATAC-seq peaks can yield insights into the transcriptional regulatory network (Miraldi et al., 2019). Previously, we made a direct imputation on the relationship between the most possible transcription factors based on the highest expression (Liu et al., 2020). Herein, we surprisingly found that the enrichment and expression of TFs in the same family (with similar domains) exhibited a distinct stage-dependent pattern. For instance, KLF4 and KLF5, and other zinc finger TFs were enriched in clusters 1, 2, and 4 NFRs associated with the expression of odontogenic genes. We had previously revealed that other zinc-finger TFs not enriched also contribute to early chromatin accessibility maintenance, such as ZEB1 (Xiao et al., 2021a) and SALL1 (Lin et al., 2021). Given the similar binding motifs of all the transcription factors belonging to the same family, it is reasonable to hypothesize that the functional redundancy among zinc-finger TF robustly maintains the odontogenic potential. Such redundancy is common in other tissues, for instance, the function of *tfap2a* and *tfap2c* in melanocyte differentiation (Li and Cornell, 2007).

In summary, the present study outlined the landscape of chromatin accessibility during odontoblast terminal differentiation, broadening our understanding of how chromatin associates and predetermine the fate of odontoblast lineage. Also, our findings could serve as a consensus for understanding dentinogenesis using dental mesenchymal stem cells. Moreover, several TF families have been revealed and are associated with chromatin accessibility in a stage-dependent manner. ATF5, for instance, promotes this process. However, a detailed functional analysis of the interaction between a specific TF or TF family and chromatin regions (such as enhancers) needs more *in vivo* validation.

## Data Availability

The datasets presented in this study can be found in online repositories. The names of the repository/repositories and accession number(s) can be found below: National Genomic Data Center, ngdc.cncb.ac.cn, CRA004359.
