# Supplementary material for: Chromatin Accessibility Predetermines Odontoblast Terminal Differentiation
Source: Front Cell Dev Biol. 2021 Nov 25;9:769193. doi: 10.3389/fcell.2021.769193 (PMC8655119; doi:10.3389/fcell.2021.769193)
Supplement: Supplementary file 1 [file DataSheet1.docx]

**Chromatin accessibility predetermines odontoblast terminal differentiation**

Qian Zhang^1#^, Zhen Huang^2#^, Huanyan Zuo^1#^, Yuxiu Lin^1^, Yao Xiao^1^, Yanan Yan^2^, Yu Cui^1^, Chujiao Lin^3^, Fei Pei^1^, Zhi Chen^1*^, Huan Liu^1,4*^

^1^The State Key Laboratory Breeding Base of Basic Science of Stomatology & Key Laboratory for Oral Biomedicine of Ministry of Education, School and Hospital of Stomatology, Wuhan University, 237 Luoyu Road, Wuhan 430079, China

^2^Fujian Key Laboratory of Developmental and Neuro Biology, College of Life Science, Fujian Normal University, Fuzhou 350108, China

^3^Division of Rheumatology, Department of Medicine, University of Massachusetts Medical School, Worcester, MA 01605, USA

^4^Department of Periodontology, School of Stomatology, Wuhan University, Wuhan 430079, China

^#^ Authors contributing equally to this article

**Supplementary Figures and Legends**

**Supplementary Figure 1**


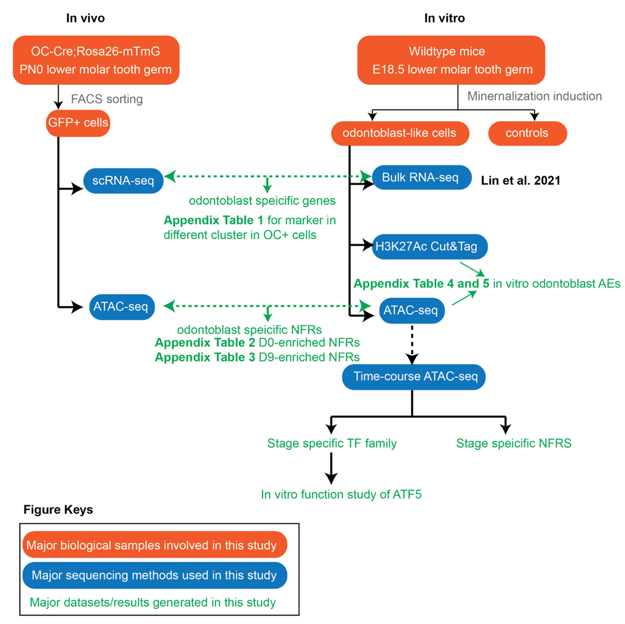


**Supplementary Figure 1. Study design**

**Supplementary Figure 2**


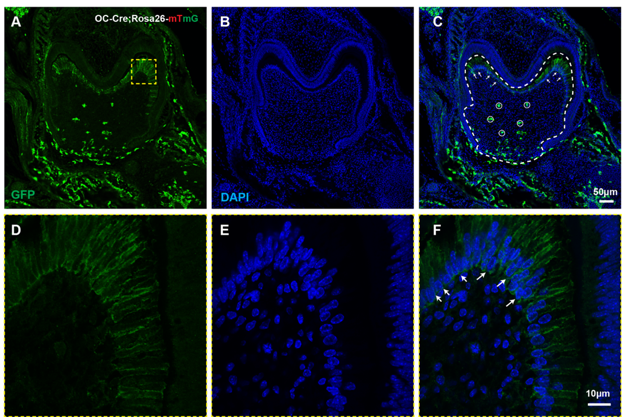


**Supplementary Figure 2. Location of OC-positive cells in PN0 OC-Cre;Rosa26-mTmG 1st lower molar.**

Immunoflouresence staining against GFP in the 1st lower molar. GFP channel (**A** and **D**), DAPI channel (**B** and **E**), merged (**C** and **F**). White circles indicate blood vessels in the tooth germ. White dash line outlines the tooth germ isolated for single-cell RNA-seq and ATAC-seq in this study.

**Supplementary Figure 3**


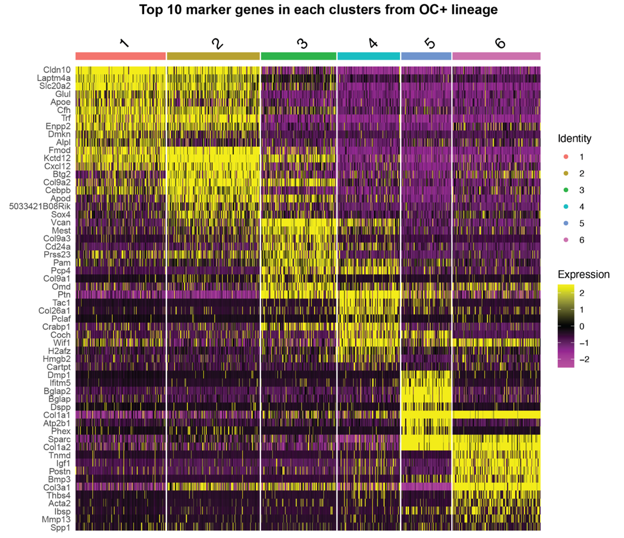


**Supplementary Figure 3. Heatmap showing top 10 enriched marker genes across 6 clusters of OC+ odontogenic cell scRNA-seq.**

**Supplementary Figure 4**


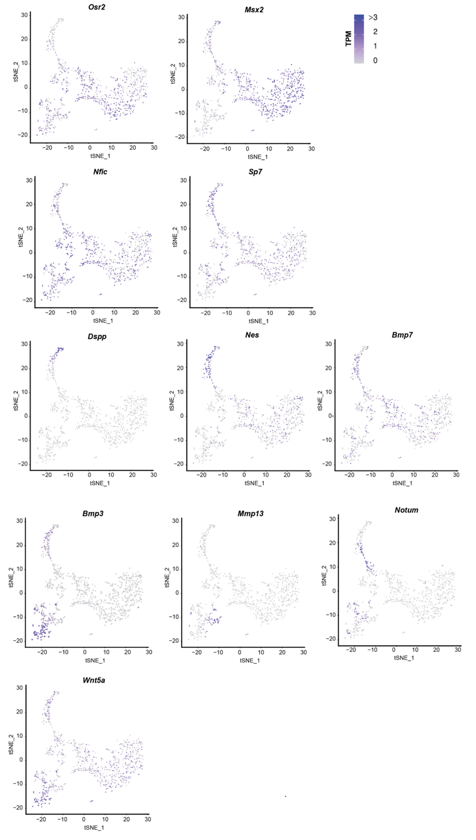


**Supplementary Figure 4. tSNE plot showing distribution of different marker genes across 6 different clusters of OC+ cell scRNA-seq**

**Supplementary Figure 5**


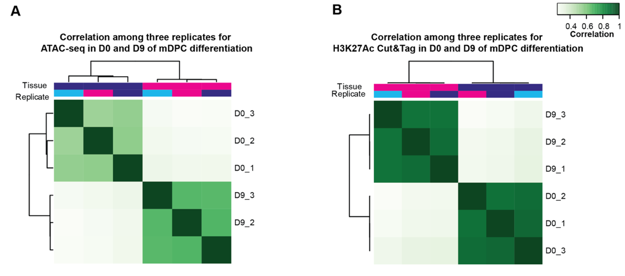


**Supplementary Figure 5. Correlation of different biological replicates for ATAC-seq and H3K27Ac Cut&Tag in D0 and D9 of mDPC differentiation.**

**Supplementary Figure 6**

**
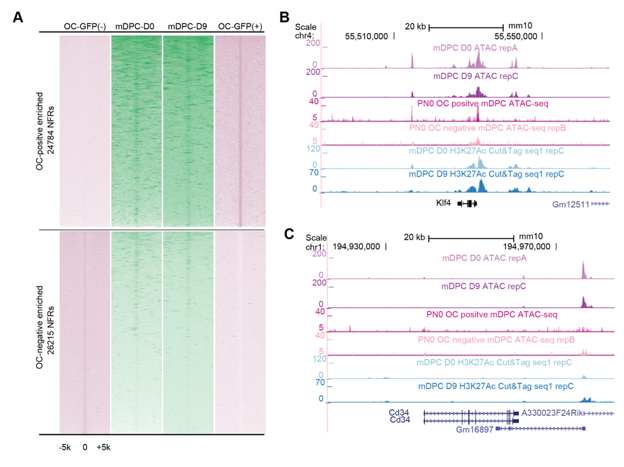
**

**Supplementary Figure 6. Integration of ATAC-seq from *in vivo* and *in vitro* odontoblast differentiation.**

**A.** Density plot showed no significant difference of ATAC-seq from mDPC-D0 and mDPC-D9 in OC-positive enriched NFRs. **B.** and **C.** UCSC genome browser view showing two examples of integrated view of *in vitro* and *in vivo* odontoblast differnetiation ATAC-seq results.

**Supplementary Figure 7**


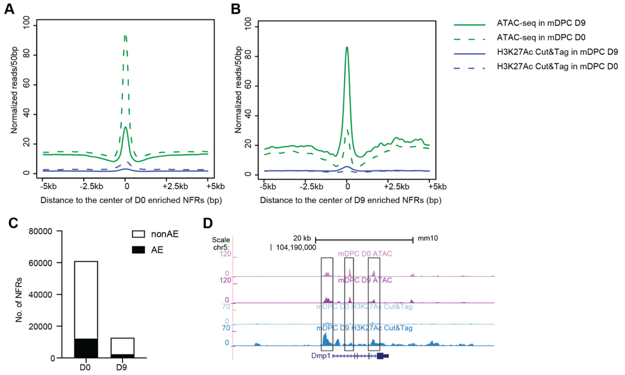


**Supplementary Figure 7. Comparison of H3K27Ac Cut&Tag and ATAC-seq in D0 and D9.**

Plot for read density of H3K27Ac Cut&Tag at D0 and D9 to the center of D0- (**A.**) and D9- (**B.**) enriched NFRs. **C.** Bargraph showing numbers of stage-specific NFRs annotated as active enhancers. **D.** UCSC genome browser view for ATAC-seq and H3K27Ac Cut&Tag near Dmp1 locus, showing some NFRs gradually lose accessibility acompanied with increased H3K27Ac modification.

**Supplementary Figure 8**


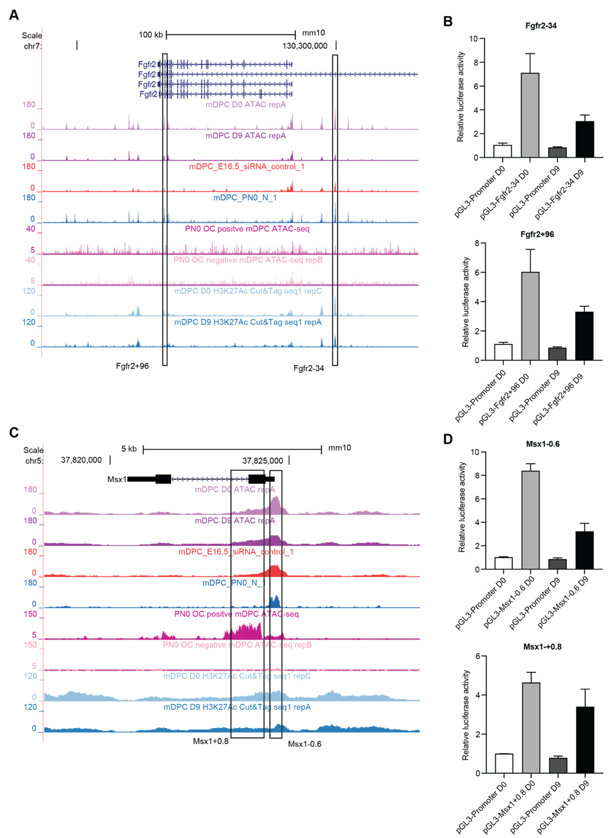


**Supplementary Figure 8. Dual luciferase assay for four selected elements enriched in D0 ATAC-seq.**

UCSC genome browser views showing the location of four selected active enhancers (black boxed) (**A.** and **C.**) Dual luciferase assay for reporter plasmids carring four selected elements transiently transfected mDPCs before and after mineralization induction (**B.** and **D.**).

**Supplementary Figure 9**


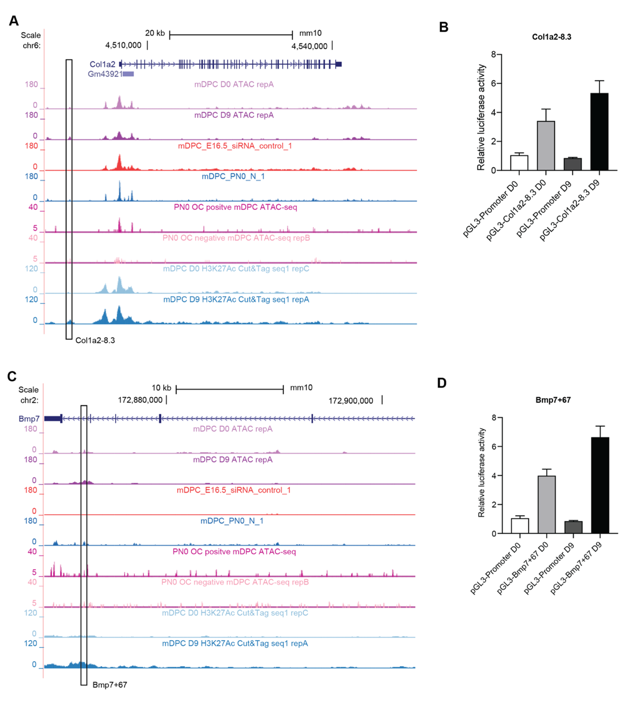


**Supplementary Figure 9. Dual luciferase assay for two selected elements enriched in D9 ATAC-seq.**

UCSC genome browser views showing the location of two selected active enhancers (black boxed) (**A.** and **C.**) Dual luciferase assay for reporter plasmids carring two selected elements transiently transfected mDPCs before and after mineralization induction (**B.** and **D.**).

**Supplementary Figure 10**


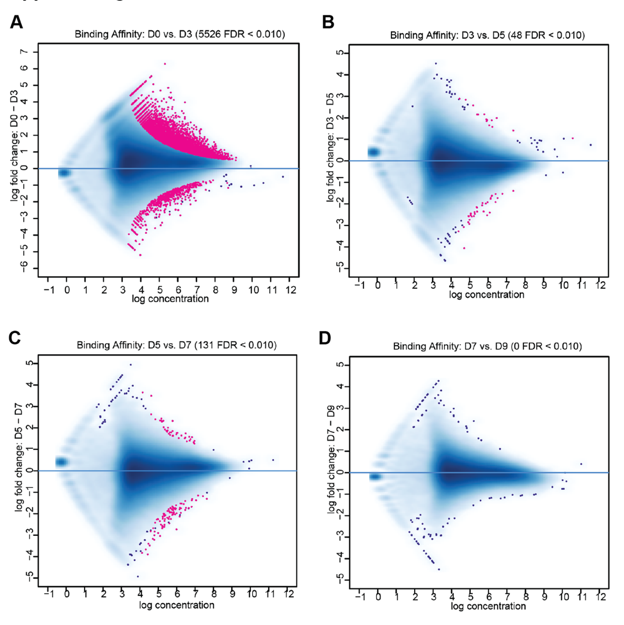


**Supplementary Figure 10. MA plot showing number and distribution of differential accessible NFRs during time-cours ATAC-seq**

**Supplementary Figure 11**


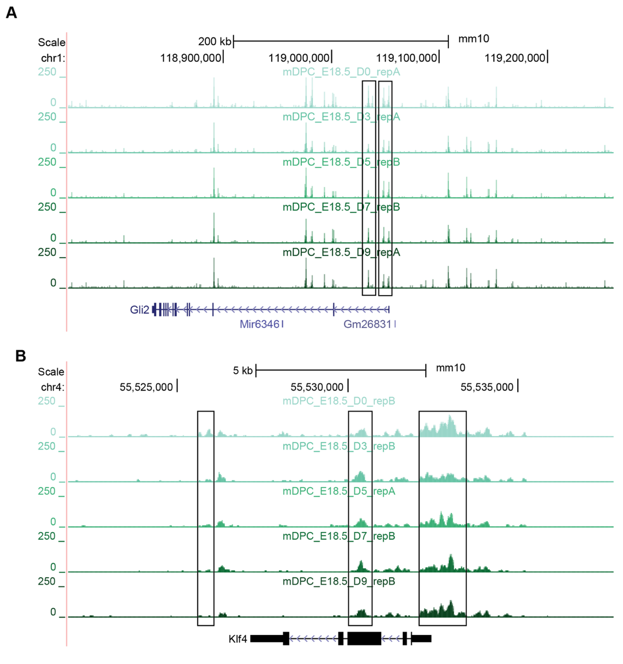


**Supplementary Figure 11. UCSC genome browser view for time-course ATAC-seq near Gli2 (an example of genes associated with cluster 1 NFRs) locus and Klf4 (an example of genes associated with cluster 2 NFRs)**

**Supplementary Figure 12**


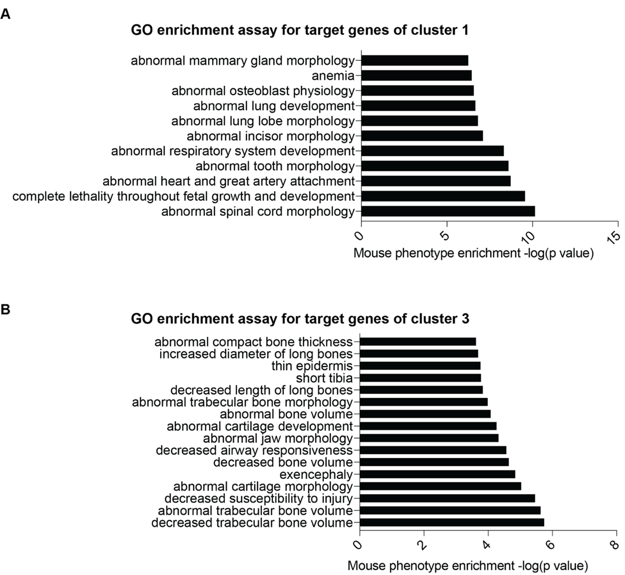


**Supplementary Figure 12. GO enrichment assay for genes associated NFRs in cluster 1 and cluster 3 in Figure 3.**

**Supplementary Figure 13**


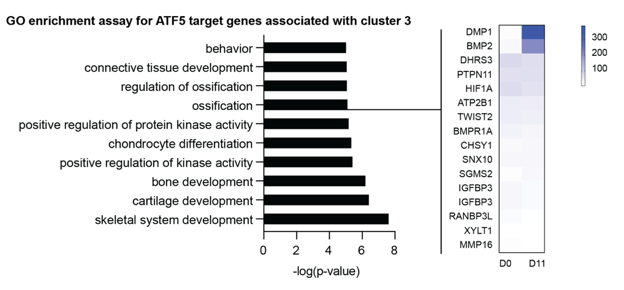


**Supplementary Figure 13. GO enrichment assay for genes associated with NFRs in cluster 3 and occupied with ATF5 motifs.** Heatmap showing average gene expression in the bulk RNA-seq results for in vitro odontoblast differentiation**.**

**Supplementary Figure 14**

**
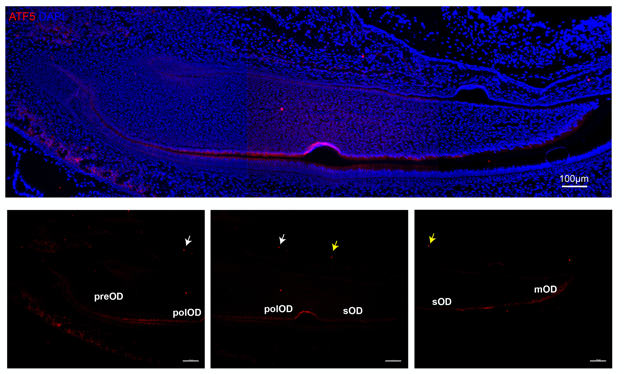
**

**Supplementary Figure 14 Immunofluorescence (IF) staining of ATF5 in PN2 murine lower incisor.** ATF5 expression in the pre-odontoblasts (preOD), polarized odontoblasts (polOD), secretory odontoblast (sOD), and mature odontoblast (mOD) were gradually increasing. Brightness of IF in different scopes were recalibrated using the overlapped auto-fluorescent particles (indicated by white and yellow arrows).

**Supplementary Figure 15**
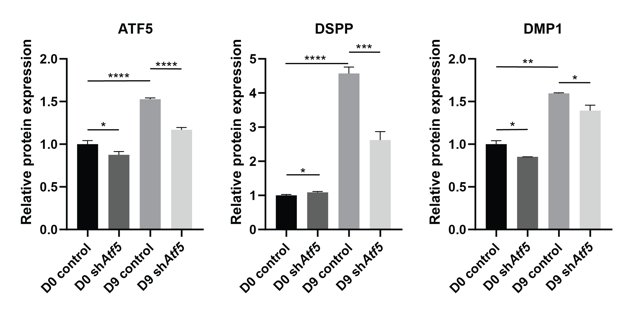


**Supplementary Figure 15. Relatively quantification of Western Blot in Figure 4A.**

**Supplementary Figure 16**


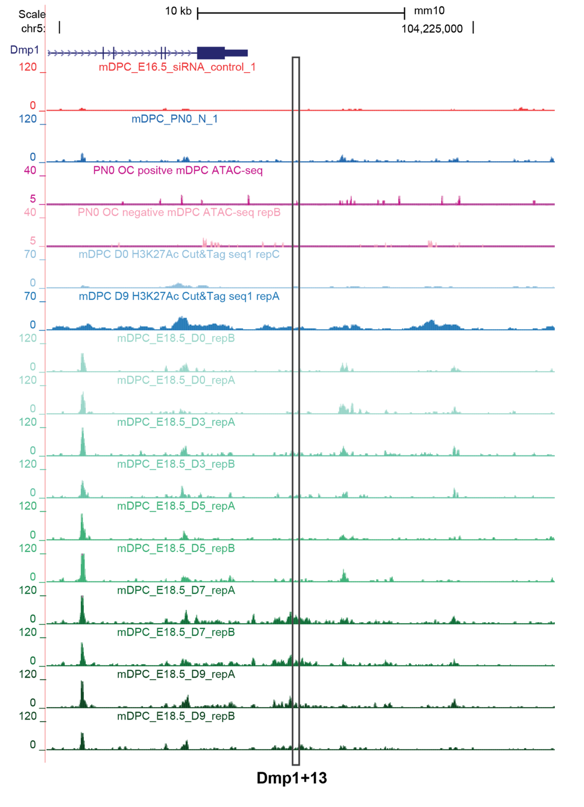


**Supplementary Figure 16. UCSC genome browser showing alignment of Dmp1+13 elements in all the ATAC-seq and H3K27Ac Cut&Tag peaks.**

**Supplementary Tables below are all deposited on github (https://github.com/Badgerliu/mDPC_epi_paper)**

**Supplementary Table 1** Marker Genes in Different Clusters of OC-positve Odontogenic Lineage from 1st Lower Molar

**Supplementary Table 2** mDPCs D0-enriched NFRs

**Supplementary Table 3** mDPCs D9-enriched NFRs

**Supplementary Table 4** mDPCs D0-enriched Active Enhancers

**Supplementary Table 5** mDPCs D9-enriched Active Enhancers
